# Pleuro-Pneumonia on Boston Ships

**Published:** 1893-02

**Authors:** Williamson Bryden

**Affiliations:** Live Stock Inspector for British Steamships at Boston, Mass.


26 Sudbury Street, Boston,
January 8, 1893.

*Editor Journal of Comparative Medicine and Veterinary Archives:*

Dear Dr.—I herewith inclose you a clipping from the Boston *Herald* of January 6th. It is an interview with me regarding eight cases of pleuro-pneumonia, reported to have been found on board of two of our latest and finest ships, the "Ottoman" and the "Michigan," on their arrival at the Port of Liverpool a few days ago:

January 6, 1893.

Those Diseased Cattle.—*Dr. Bryden Explains Cases of Pleuro-Pneumonia on Two Boston Ships.*—A dispatch from Ottowa, Ont., stated yesterday that Sir Charles Tupper had notified the Department of Agriculture of the arrival at Liverpool of two steamers from Boston, the Ottoman and Michigan, with cattle on board, eight of which were found to be affected with pleuro-pneumonia.

Dr. William Bryden, who inspected the cattle on those ships before they went over, said to a Herald reporter this morning:

"Notice that the dispatch says nothing about the cases being contagious pleuro-pneumonia. The cattle on the Ottoman and Michigan were the average western cattle, and there was nothing about them to indicate disease of any kind.

"There is a difference between different schools as to whether we have contagious pleuro-pneumonia or a simple accidental or sporadic pneumonia in cattle.

"The veterinarians of the British Agricultural Department do not, on the arrival of cattle, take time to discriminate between the two, and so take the benefit of the doubt, and call a number of lung lesions, accidentally caused, true contagious pleuro-pneumonia, which I regard as very unfair.

"I have been inspector for the ships many years, and have had the case come before me before. It is the same old misunderstanding between veterinarians.

"These cases are quite parallel with the cases recently reported from Canada, about which there was so much said at the time.

"In my 14 years' experience in inspecting 25,000 to 100,000 head of cattle a year, I have never seen a case of contagious pleuro-pneumonia in those shipped."

That the veterinarians of the British Agricultural Department are *sincere* in their *course* there can be little doubt, from the fact that it does not affect the traffic in live stock from the United States, only in so far as it *delays* the admission to Great Britain of "stockers," or lean cattle. With Canada the case is different. Her cattle have been not only *set back* to the status of animals from a scheduled country, but several cargoes have been sacrificed on account of a few suspicious cases having been found among them, being lean they were unfit to kill, and being suspected of a contagious disease which, according to Act of Parliament, demanded their slaughter, it had to be carried out. This is a feature of sanitary laws relating to certain contagious diseases of animals in many countries, and it was carried out with as much thoroughness, and as arbitrarily, against their own people as against those of other nations.

The disposition frequently manifested by Secretary Rusk and his friends to attribute unfairness on the part of the British Agricultural Department, and hostility to the United States, as their motive for condemning her cattle is therefore an unjust and illogical conclusion, so long as her *own* colonies are treated with even greater severity than the United States.

The country that adheres to its laws so consistently that it spares not its own people is certainly entitled to respect, however much mistakes of officials are to be deplored. America is indebted to Prof. Williams, of Edinburgh, more than to any one else. He defended her cattle, and practically demonstrated the differences between the *lung lesions of her cattle* and those affected with *contagious lung disease*, when every one was against him.

The veterinary profession is undergoing an examintion, at the present time, into its methods of dealing with contagious animal diseases in the past, which is liable to expose an amount of ignorance, heartless waste, and extravagance, that cannot be otherwise than terribly humiliating.

If the veterinarians of the British Agricultural Department are *wrong* in the present dispute they cannot escape censure, not for willful hostility to the United States, but for ignorance of the true character of a disease that it was criminal for them not to know. Principal Williams having proved it to them nearly a dozen years ago with the same class of animals.

The United States narrowly escaped a similar misfortune when, two or three years ago, it was proclaimed from *a supposed reliable source* "that from 35 to 50 per cent. of the cattle population of Massachusetts were tuberculous."

Had a sanitary organization been formed of men whose notions of the disease and its prevalence harmonized with such estimates, it would have cost the State as much as another war, for the country is full of men ready to perform just such heartless work.

WILLIAMSON BRYDEN,

*Live Stock Inspector for British Steamships at Boston, Mass.*

